# Review of Atypical and Anaplastic Meningiomas: Classification, Molecular Biology, and Management

**DOI:** 10.3389/fonc.2020.565582

**Published:** 2020-11-20

**Authors:** Taylor Anne Wilson, Lei Huang, Dinesh Ramanathan, Miguel Lopez-Gonzalez, Promod Pillai, Kenneth De Los Reyes, Muhammad Kumal, Warren Boling

**Affiliations:** Loma Linda University, Loma Linda, CA, United States

**Keywords:** atypical meningioma, anaplastic meningioma, high grade meningiomas (HGMs), WHO grade II meningioma, WHO grade III meningioma

## Abstract

Although the majority of meningiomas are slow-growing and benign, atypical and anaplastic meningiomas behave aggressively with a penchant for recurrence. Standard of care includes surgical resection followed by adjuvant radiation in anaplastic and partially resected atypical meningiomas; however, the role of adjuvant radiation for incompletely resected atypical meningiomas remains debated. Despite maximum treatment, atypical, and anaplastic meningiomas have a strong proclivity for recurrence. Accumulating mutations over time, recurrent tumors behave more aggressively and often become refractory or no longer amenable to further surgical resection or radiation. Chemotherapy and other medical therapies are available as salvage treatment once standard options are exhausted; however, efficacy of these agents remains limited. This review discusses the risk factors, classification, and molecular biology of meningiomas as well as the current management strategies, novel therapeutic approaches, and future directions for managing atypical and anaplastic meningiomas.

## Introduction

Harvey Cushing, in his 1922 publication, suggested the term *meningioma* to describe tumors arising from the pachymeningeal coverings of the brain and spinal cord, and he hypothesized these lesions arose from the arachnoid cap cells (1–3). Meningiomas are the most common primary intracranial tumors with an incidence of 2.3–8.3 in 100,000 (4–9). Although most meningiomas are benign (80%) and slow-growing, atypical (15–20%) and anaplastic (1–3%) meningiomas are more aggressive with a proclivity for recurrence, worse clinical outcomes, and higher disease-specific mortality (7, 10–13). Ideal management of higher grade meningiomas remains debated, specifically concerning use of adjuvant radiation in patients following complete resection of atypical meningiomas. Furthermore, recurrent meningiomas often become refractory to standard surgical and radiation therapies, which makes management challenging. Chemotherapy and other systemic medical therapies are reserved as salvage therapies in these patients; however, they have shown limited success with a few medical treatments demonstrating marginal clinical benefit. Accurate risk stratification and tumor classification are critical in identifying patients at risk for recurrence and tailoring subsequent management. Furthermore, advancements in understanding the pathophysiology and molecular genetics of meningiomas is critical for improving risk stratification, predicting prognosis and recurrence, and designing novel treatments for these patients (14–16). In this review, we will discuss the risk factors, classification, molecular biology, and current management strategies as well as novel therapeutic approaches and future directions for managing patients with atypical and anaplastic meningiomas.

## Risk Factors

Age, male sex, and prior cranial ionizing radiation are risk factors for high grade meningiomas. The incidence of meningiomas increases with age, peaking around the 6th and 7th decades, but high grade meningiomas have a lower median age of diagnosis than benign meningiomas. Whereas benign meningiomas have a much higher incidence in females, atypical and anaplastic meningiomas occur almost twice as often in males (17, 18). Approximately 70–80% of meningiomas express progesterone receptors, and to a lesser extent, estrogen receptors, which corroborates the theory of a hormonal component to growth and provides an explanation for the higher incidence in females. High levels of progesterone receptors are associated with favorable prognosis, whereas meningiomas with loss or absence of progesterone receptors tend to be more aggressive with increased rates of recurrence (9, 19–22).

Meningiomas are very rare in children, but those with a history of cranial ionizing radiation are reported to have a 6–10 times increased relative risk of developing a meningioma with an elevated risk of atypical or anaplastic features (23). The strongest increase in incidence of meningioma occurrence has been identified after craniospinal radiation for the treatment of childhood acute lymphoblastic leukemia (ALL) and in individuals who received low dose radiation for the treatment of cranial tinea capitis (24). In atomic bomb survivors a significant dose related increase in intracranial tumors, including meningiomas (25). The association of ionizing radiation to meningioma development has been clearly established in individuals who received low dose radiation to the head for the treatment of tinea capitis (26). Cranial radiation on the order of 1–2 Gy significantly increased the risk of meningioma and glioma with the highest relative risk of development of nerve sheath tumors. One common theme among all the reports of secondary meningiomas is that the tumor typically occurred several decades after the radiation exposure.

Atypical and anaplastic meningiomas occur more frequently over the cerebral convexities than at the skull base. Additionally, when these high grade meningiomas occur at the skull base, they have lower recurrence rates and better overall prognosis than similar tumors found over the convexities (27, 28).

There are several inherited genetic syndromes that predispose patients to developing a meningioma. Neurofibromatosis 2 (NF2) is the most common and well-known. The neurofibromin 2 gene, also known as merlin, is located on chromosome 22q, and deletion or any other mutation at this site is associated with meningioma development (29). Other syndromes associated with meningiomas include multiple endocrine neoplasia (MEN) type 1 and von Hippel-Lindau (30).

## Classification

The WHO grading system classifies meningiomas into grade I (benign), grade II (atypical), and grade III (anaplastic) based on histopathological features associated with tumor aggressiveness and tendency for recurrence (10, 11). The 1993 WHO classification was the first effort of the WHO to organize meningiomas by tumor grade, but there was criticism over this edition due to vague criteria, which led to high interobserver variability in reporting tumor grade. Since the 2000 edition, the WHO classification system has remained largely unchanged with the exception of brain invasion, as these newer editions have more objective criteria with less variation in classifying tumors among physicians. In the 2000 WHO classification, brain invasion was not a criterion for grade II or grade III meningiomas; however, later studies have shown brain invasion to be associated with aggressive behavior and increased risk of recurrence. The 2007 WHO classification was therefore revised to include brain invasion as an independent criterion for grade II (atypical) meningiomas (10). Since this change, the proportion of atypical meningiomas has increased from ~7 to 15–20% (13, 31, 32). In the most recently published 2016 WHO classification, there were no further modifications to grading criteria (11). Criteria for grade II and grade III meningiomas across the different WHO editions are shown (Table 1).

**Table 1:** WHO classifications for Grade II and Grade III meningiomas by year.

**Year of Classification**	**WHO Grade II**	**WHO Grade III**
1993	Several of the following • Frequent mitoses • Hypercellularity • Small cells with high nuclear to cell ratio • Prominent nucleoli • Small cells with high nuclear to cell ratio	Histological features of frank malignancy far in excess of the abnormalities noted in atypical meningiomas
2000	Mitotic rate 4–19 per 10 HPF OR Three or more of the following • Hypercellularity • Small cells with high nuclear to cell ratio • Prominent nucleoli • Patternless sheet-like growth • Spontaneous or geographic foci of necrosis	High mitotic rate >20 per 10 HPF OR Frank anaplasia with loss of meningothelial differentiation, often resembling carcinoma, sarcoma, or melanoma
2007/2016	Mitotic rate 4–19 per 10 HPF OR Brain invasion OR Three or more of the following • Hypercellularity • Small cells with high nuclear to cell ratio • Prominent nucleoli • Patternless sheet-like growth • Spontaneous or geographic foci of necrosis OR Histologic subtypes: Clear cell and choroid	High mitotic rate >20 per 10 HPF OR Frank anaplasia with loss of meningothelial differentiation, often resembling carcinoma, sarcoma, or melanoma OR Histologic subtypes: Papillary and rhabdoid

* *HPF: High-power field*.

**Figure 1:**
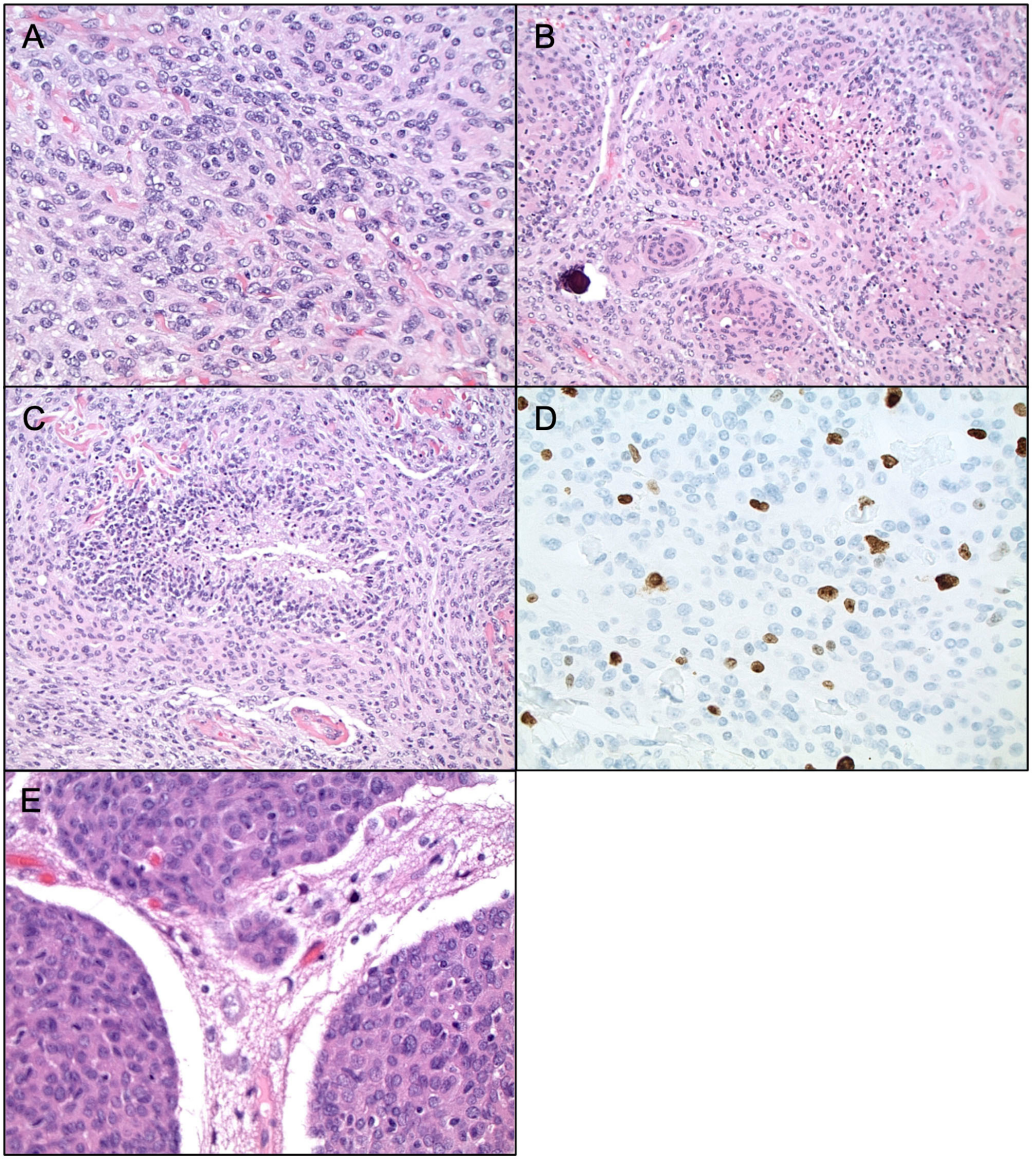
Histopathology of Atypical Meningiomas. Atypical meningioma (WHO grade II). **(A)** H&E staining, ×400 magnification, demonstrating cell sheeting. **(B)** H&E staining, ×200 magnification, demonstrating whorls, and early focus of degeneration. **(C)** H&E staining, ×200 magnification, demonstrating necrosis. **(D)** Ki67 staining, ×400 magnification, demonstrating proliferation indices. **(E)** H&E staining, ×400 magnification, demonstrating brain invasion.

**Figure 2:**
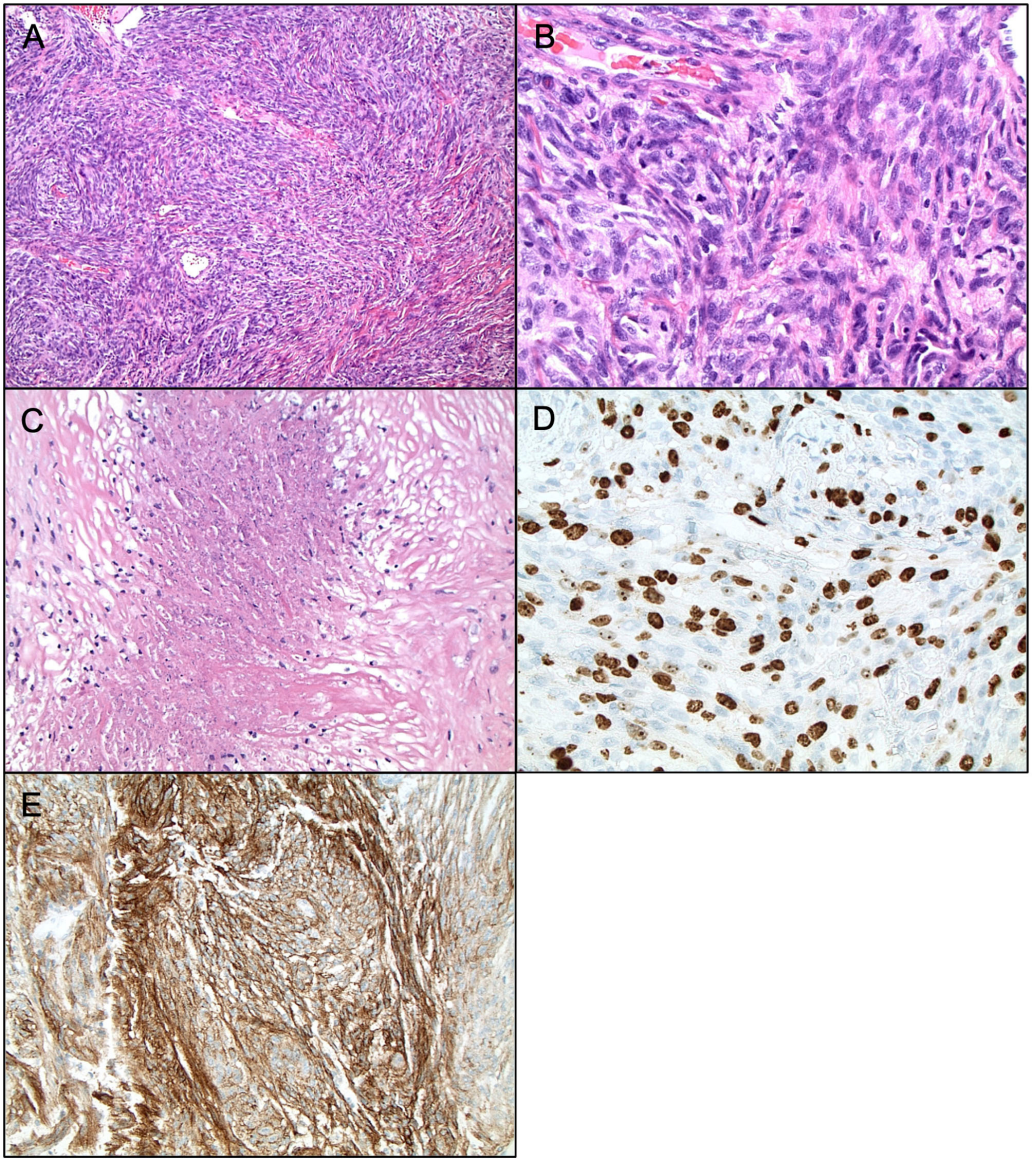
Histopathology of Anaplastic Meningiomas. Anaplastic meningioma (WHO grade III). **(A)** H&E staining, ×200 magnification. **(B)** H&E staining, ×400 magnification, demonstrating mitoses >20 per high powerfield. **(C)** H&E staining, ×200 magnification, demonstrating frank necrosis. **(D)** Ki67 staining, ×400 magnification, demonstrating proliferation indices. **(E)** EMA staining, ×200 magnification.

Currently, Grade II (atypical) meningiomas are characterized by increased mitoses (4–19 mitotic figures per 10 high power microscope fields), brain invasion, or presence of three of the following five histologic features: hypercellularity, small cells with high nuclear to cellular ratio, prominent nucleoli, patternless sheet-like growth, and spontaneous or geographic foci of necrosis (Figures 1A–E). The clear cell and chordoid subtypes are also considered atypical (10, 11, 33). Grade III (anaplastic) meningiomas exhibit histologic features of overt malignancy, including high mitotic activity (20 or more mitotic figures per 10 high power microscope fields), frank anaplasia with focal, or diffuse loss of meningothelial differentiation, and their cytology often resembles carcinoma, sarcoma, or melanoma (Figures 2A–E). The rhabdoid and papillary subtypes are also classified as anaplastic (10, 11, 33). Metastases are not common with meningiomas, but they can occur.

The WHO classification is an important prognostic tool, but it has several limitations. First, despite revisions, the grading criteria remain somewhat vague, and studies have demonstrated inter-observer differences in applying these criteria. Additionally, the WHO grading system is based solely on histologic criteria, and unlike many other CNS tumors, objective molecular and genetic data is not used in classification of meningiomas. Furthermore, there is substantial within grade variation among tumors with studies reporting indolent behavior with no recurrence in up to 71 and 50% of atypical and anaplastic meningiomas, respectively (34–36). Thus, the WHO classification is inadequate for entirely predicting tumor aggressiveness, recurrence, and prognosis, and alternative methods are required for more adequate risk stratification (37).

In addition to the aforementioned WHO criteria, the mouse intestinal bacteria 1 (MIB-1) proliferation index is a histopathological biomarker that is associated with higher recurrence rates in meningiomas (38). A higher MIB-1 index is associated with worse prognosis with one study reporting MIB-1 indices of 1.9, 4.5, and 11.7% in benign, atypical, and anaplastic meningiomas, respectively (39–41). Studies have shown that the MIB-1 proliferation index is a more sensitive proliferation marker than mitotic rate. The MIB-proliferation index has been most commonly used as an adjunct to WHO criteria, and it is particularly useful in borderline cases for determining tumor grade and prognosis (42, 43).

## Molecular Biology

Advancements in understanding the pathophysiology and molecular biology of meningiomas is critical for improving risk stratification, predicting prognosis and recurrence, and designing novel treatments for these patients. As molecular analyses of meningiomas continue to evolve, several cytogenetic, genomic, epigenetic, and expression alterations associated with tumor aggressiveness and proclivity for recurrence have been identified as potential biomarkers to enhance diagnosis and risk stratification as well as serve as sites to target new therapies.

### Cytogenetics and Genomics

Genomic instability is associated with tumor aggressiveness, and karyotype abnormalities are observed in progressively increasing frequency as a meningioma becomes more aggressive. Several cytogenetic abnormalities have been identified in meningiomas. As mentioned briefly above, the most common cytogenetic aberration observed in meningiomas is deletion or loss of genetic loci containing the neurofibromin 2 (NF2) gene on chromosome 22q. This alteration occurs in 40–60% of meningiomas. This gene encodes a tumor suppressor protein, merlin, involved in regulating activation of the mTOR pathway. Presence of this mutation is predictive of higher risk of recurrence. Meningiomas with NF2 mutations have a proclivity for the cerebral hemispheres (44).

Loss of genetic loci at chromosome 1p is the second most common aberration, and this is oftentimes seen in association with chromosome 22q mutations. Mutation of genetic loci within the Telomerase Reverse Transcriptase (TERT) promotor segment is observed in 6% of meningiomas, but this mutation occurs almost exclusively with concurrent chromosome 22q alterations, and the addition of a TERT promotor mutation is predictive of increased tumor aggressiveness and likelihood of recurrence. Other, less common, cytogenetic abnormalities associated with tumor aggressiveness and recurrence include loss at 6q, 9p, 10p, 10q, 14q, and 18q and gain at 17q and 20q (45–47).

Whereas these abnormalities are rare in benign meningiomas, they are observed frequently in atypical and anaplastic meningiomas (48, 49). Thus, accumulation of genetic aberrations increases progressively with higher tumor grade, and increasing frequency of cytogenetic alterations is associated with higher rates of recurrence and shorter progression free survival times (50, 51). Furthermore, evidence in ongoing research suggests that genetic profiles may vary by meningioma location (52).

### Epigenetics

Through whole genome analysis, global DNA methylation profiling has demonstrated higher levels of methylation are associated with increased tumor aggressiveness and risk of recurrence. DNA methylation is an epigenetic change hypothesized to contribute to genomic instability by silencing genes involved with DNA repair and control of cell cycling. Evidence suggests methylation status may predict tumor behavior more accurately than the current WHO classification, and DNA methylation status has been proposed as an alternate classification system for meningiomas (16). However, DNA methylation profiling is costly, which may limit its utility.

Recent studies have begun to investigate epigenetic modification on the level of histones with particular focus on H3K27 trimethylation (H3K27me3). Using immunohistochemistry, one study found that meningiomas absent of H3K27me3 staining were associated with significantly higher risk of progression. Furthermore, H3K27me3-negative meningiomas were associated with DNA methylation patterns observed in more aggressive meningiomas, and there was a proportionally higher percentage of NF2 mutations among H3K27me3-negative meningiomas. This study found that H3K27me3 may play a role in risk stratification, especially in meningiomas at the border of WHO I and II; however, it is less useful in grade III meningiomas (53).

### Protein Expression

Alterations in protein expressions are seen in meningiomas. Several growth factors, including vascular endothelial growth factor (VEGF), platelet-derived growth factor (PDGF), and epidermal growth factor (EGF), and their associated receptors are overexpressed in meningiomas, which stimulates tumor growth and progression in such tumors. Hormonal dysregulation occurs frequently in meningiomas. Absence of progesterone receptors is associated with increased tumor aggressiveness and recurrence, and overexpression of estrogen, somatostatin, and prolactin receptors are associated with increased proliferative activity of meningiomas (19, 54). Many of these growth factors and hormones are also overexpressed in other tumors and are the target of several new targeted therapies.

Another more recently discovered that inactivation of the breast cancer (BRCA)1-associated protein-1 tumor suppressor gene (BAP1) is found within a subgroup of rhabdoid meningiomas and may be assessed with immunohistochemistry. Loss of expression is associated with shorter time to recurrence and worse prognosis. Interestingly, a subgroup of patients with loss of expression of BAP1 have associated BRCA1 germline mutations, suggesting that patients with this mutation are also at increased risk of rhabdoid meningiomas (55).

### Immunotherapy

Meningiomas and their associated microenvironment are associated with a local immune response, and analysis of immune cell infiltrate has revealed potential biomarkers and targets for immunotherapy (56). Following encouraging results in other tumors, immune checkpoint inhibitors are being explored for treatment of meningiomas (57). Under physiologic conditions, immune checkpoints modulate the immune response and prevent autoimmunity; however, meningiomas and other tumors also utilize these checkpoints to evade immune system detection and create an immunosuppressed microenvironment (57). Programmed-death 1 (PD-1) and its ligand, (PD-L1), function as part of the immune checkpoint pathway that regulates T cell lymphocytes, and its expression in meningiomas is correlated with higher tumor grade and aggressiveness (56, 58, 59). Currently, several trials of PD-1 and PD-L1 antibody-mediated inhibition in meningiomas are underway (57).

## Surgical Resection

The primary treatment for atypical and anaplastic meningiomas is surgical resection. Small, asymptomatic meningiomas that are presumably benign may be monitored or treated with radiation, but these meningiomas are out of the scope of this paper. In 1957, Donald Simpson described this strong association between extent of recurrence (60). He classified extent of resection into five categories (Table 2). Generally, Simpson Grades I–III are considered gross total resection (GTR), and Simpson Grades IV–V constitute subtotal resection (13, 34, 61, 62). Recently, a sixth category, Grade 0, has been proposed in which there is complete tumor removal plus an additional 2–3cm from tumor insertion site with good results (63).

**Table 2:** Simpson grading for extent of meningioma resection.

**Simpson Grade**	**Description**
Grade 0	Complete tumor removal, plus removal of an additional 2–3 cm from the tumor insertion site
Grade I	Complete tumor removal, including any dural attachments or abnormal bone
Grade II	Complete tumor removal with coagulation of dural attachment
Grade III	Complete tumor removal without resection or coagulation of its dural attachment
Grade IV	Partial tumor removal
Grade V	Biopsy only

Simpson grading remains the standard method for describing surgical resection, and it is determined by the neurosurgeon's assessment and, more recently, postoperative imaging. The extent of resection is the most important modifiable predictor of local control and progression free survival, independent of tumor grade and other prognostic factors (30, 60, 64). Thus, the goal of surgery, when feasible, is GTR; however, tumor location, involvement of nearby neurovascular structures, or brain invasion may limit the extent of resection, in which case maximum safe resection is appropriate.

Prognosis is strongly related to the histopathological grade and extent of resection. Recurrence is utilized to describe patients whose meningioma returns despite complete surgical resection. Although there is no consensus on definition, progression refers to growth of residual tumor in patients with incompletely resected tumors. Furthermore, progression is also applied for meningiomas that transform from a lower to a higher-grade tumor. Following complete resection, the 5-year recurrence rate is 29–58% for atypical and 72–94% for anaplastic meningiomas (12, 30, 64) The 5-year risk of progression for incompletely resected meningiomas is as high as 83–100% (30, 65) Specifically, the 5-year survival rates are 78–91% and 41–65% for atypical and anaplastic meningiomas, respectively, and the 10-years survival rates decrease to 53% in atypical and 0% in anaplastic (34, 61, 65–67).

Multiple factors, including neurosurgeon preference, tumor size and location, extent of dural attachment, and relationship to surrounding neurovascular structures influence surgical approach. Ideally, the approach is wide enough to expose enough of the meningioma, its dural attachment, and surrounding structures to allow disruption of blood supply while simultaneously minimizing brain retraction and manipulation of critical structures to reduce procedure-related morbidity (68).

Over the past several decades, considerable advancements in surgical technologies, including the operating microscope, improved neuroimaging, image-guided neuronavigation systems, intraoperative neurophysiological monitoring, ultrasonic aspiration devise, and endovascular embolization techniques, have revolutionized modern neurosurgery improving the safety of surgery (69, 70). Introduced in the 1970s, the modern operating microscope and refinement of microsurgical technique significantly enhanced the neurosurgeons ability to carefully dissect meningiomas (71, 72). Furthermore, in the late 1980s, new technology with spatially accurate neuroimaging, computer-assisted imaging systems, and three dimensional digitizers allowed integration in image space with operative space and led to development of more modern, frameless stereotactic image-guided navigation (69). Typically, unless contraindicated, contrast-enhanced CT or MRI imaging is used, but these images can be fused with additional studies, such as PET or functional MRI, to improve visualization of structures of interest (73). With contemporary neuronavigation systems, neurosurgeons are able to preoperatively plan surgeries and explore alternate approaches. Furthermore, using multiplanar imaging, neuronavigation provides real-time intraoperative guidance and data regarding the location and orientation of surgical instruments in relation to nearby structures (69, 70, 74).

Ultrasonic aspiration devices are another valuable tool for resecting meningiomas, especially larger ones. These devices are used to internally debulk meningiomas, which helps avoid damage to adjacent brain and other neurovascular structures during tumor dissection. Furthermore, through tissue selection, the ultrasonic transducer spares vital surrounding neurovascular structures (75–77).

Moreover, as endovascular techniques advance, preoperative embolization has been increasingly used to facilitate meningioma resection and decrease intraoperative blood loss, especially in select patients with giant convexity meningiomas or petroclival meningiomas in which the feeding arteries may be less accessible during surgery. A systematic review of preoperative embolization for meningiomas by Shah et al. (78), reported that liquid embolic agents were preferable to particle agents as liquid agents demonstrated deeper penetration into the tumor vessels and had a smaller risk of hemorrhage.

In addition to enhanced safety, these innovations improve tumor access, debulking, and extent of resection, especially in meningiomas that were once considered unresectable or partially resectable (52). Furthermore, these technologies are associated with decreased blood loss, reduced operative times, fewer complications, and, accordingly, shorter ICU and overall hospital length of stays (73, 74, 79, 80). Moreover, many neurosurgeons report an enhanced appreciation of anatomy and increased perception of safety (74, 80). Most importantly, however, technology does not replace the neurosurgeon's knowledge and skills, and it is critical for neurosurgeons to be aware of limitations and potential for error, especially regarding neuronavigation systems.

## Radiation

Radiation is an effective and generally well-tolerated treatment for meningiomas. Based on evidence in the literature, adjuvant radiation is usually recommended for atypical meningiomas following incomplete resection, for anaplastic meningiomas regardless of the extent of resection, and for recurrent meningiomas (81–86). However, in patients with completely resected atypical meningiomas, the role of adjuvant radiosurgery remains undefined, and there remains considerable debate regarding optimal management of these patients with treatment decisions varying based upon physician preference (5, 31, 34, 64, 85, 87–89). Advocates argue that adjuvant radiation reduces the risk of recurrence, increases time to recurrence and tumor burden in those who develop recurrence, and improves disease-specific survival (34, 61, 64, 84, 85, 88–92). Opponents, however, argue that adjuvant radiation does not reduce risk of recurrence, and the costs and potential harm associated with possibly unnecessary radiation outweighs any benefits (32, 64, 65, 81, 93).

Regarding the literature, there is a paucity of high-quality evidence regarding adjuvant radiation for patients with completely resected atypical meningiomas. The majority of studies are small retrospective with low power and inconsistent results. Although several of these studies reported lower recurrence rates with adjuvant radiation, many were unable to demonstrate statistical significance (31, 34, 64, 85, 89). Other studies, however, showed no difference in recurrence rates with adjuvant radiation vs. actively monitoring (17, 31, 62, 81, 94). Few studies report long term follow up of 10 years of more, but some evidence suggests the benefits of adjuvant radiation may be more significant in the long term as median recurrence rates for atypical meningiomas are longer than anaplastic meningiomas (95, 96). The main findings regarding efficacy of post-surgery adjuvant radiation were summarized in Table 3.

**Table 3:** Summary of the main studies regarding efficacy of adjunctive radiotherapy in atypical (Grade II) meningiomas.

**Author**	**Study type**	**WHO**	**Patients number**	**ART Regimen**	**Outcome of ART vs no ART**
Mair et al. (31)	Retrospective	2000	114 patients (*n* = 84 no ART; *n* = 30 ART)	Average dose of 51.8 Gy in 28 fractions over 6 weeks	ART did not reduce overall tumor recurrence following first-time surgery. Significant benefit was evident if excluded the patients who had undergone postoperative stereotactic radiosurgery for a tumor remnant (and no radiotherapy) from analysis.
Aghi et al. (34)	Retrospective	2004	108 (*n* = 70 no ART; *n* = 38 ART, of which 8 received ART after initial GTR; 30 with recurrent tumor)	8 patients after CRT, received fractionated stereotactic radiotherapy at an average dose of 60.2 Gy in 1.5–1.8-Gy fractions. In 30 patients with recurrent tumors, 14 received fractionated stereotactic radiotherapy at mean dose of 55 Gy and 16 received single-fraction stereotactic radiosurgery at mean marginal dose of 18.0 Gy	None of these 8 patients experienced tumor recurrence, but there was no statistical difference in recurrence between irradiated and nonirradiated patient. Most recurrences occurred within 5 years after resection. One-third of patients with recurrence died of their disease despite irradiation or chemotherapy at the time of recurrence.
Graffeo et al. (64)	Retrospective with meta-analysis with additional 9 retrospective studies	2016	69 patients (*n* = 61 no ART; *n* = 8 ART)	A median dose of 5,400 cGy over median 30 fractions	Overall recurrence at time of last follow-up was 25% after observation and 38% after RT, with median times to recurrence of 176 and 101 months, respectively. At 5 years, PFS was 79% after observation and 88% after RT; however, OS was 89% after observation and 83% after RT. Thus, preemptive ART has no significant advantage on either recurrence or survival.
Hasan et al. (84)	Meta-analysis. Including 14 retrospective studies	Not specified	757 patients (*n* = 549 no ART; *n* = 208 ART)	A median dose of 54 Gy	The crude recurrence rate was twice as high in GTR than GTR with ART (33.7 vs. 15%, *P* = 0.005). The 1-year local control rate was 90% for GTR and 97% for GTR with A RT (OR = 3.36, *P* = 0.11). The median 5-year local control rate was 62% for GTR and 73% for GTR with ART, respectively (OR = 1.71, *P* = 0.06). The 5-year overall survival for each group was 90%, which was not were not significantly different (OR = 0.97, *P* = 0.95). Radiation-related toxicity was <10%, at a median follow-up of 42 months.
Park et al. (85)	Retrospective	2000/2007	83 patients (*n* = 56 no ART, *n* = 27 ART)	A median dose of 61.2 Gy over 7 weeks with photon	ART led to lower local tumor progression.
Komotar et al. (89)	Retrospective	Not specified	45 patients (*n* = 32 no ART; *n* = 13 ART)	A median dose of 59.4 Gy in daily fractions of 180 or 200 cGy and completed over a median of 6 weeks	There were no recurrences in 12 (92.3%) of 13 ART patients. No other factors were significantly associated with recurrence in univariate or multivariate analyses.
Stessin et al. (94)	Retrospective	2000	657 patients (*n* = 413 no ART; *n* = 244 ART)	Not specified	Patients with Grade III disease were 41.9% more likely to receive ART than that of Grade II meningioma, 36.7% more likely to receive it after subtotal resection (95% CI 0.58–3.26). Controlling for grade, extent of resection, size and anatomical location of the tumor, year of diagnosis, race, age, and sex, ART did not have a survival benefit (HR 1.492; 95% CI 0.827–2.692)
Jo et al. (96)	Retrospective	2000	35 patients (*n* = 13 no ART; *n* = 21 ART)	Not specified	The median interval to recurrence was 17 months (range = 5–46 months) for the patients who underwent surgery alone, and 39 months (range = 13–97 months) for the patients in ART group. ART following initial incomplete surgical resection was crucial for long-term management.
Jenkinson et al. (97)	Prospective	2000	190 patients will be enrolled (comparing no ART vs. ART)	60 Gy in 30 fractions over 6 weeks.	Results not reported yet

*ART, Adjunctive Radiotherapy; GTR, Gross total resection*.

Although the specifics vary depending on organ system and some of the criteria are somewhat vague, radiation-induced toxicities are generally graded from 1 to 5: grade I is mild symptoms, grade II is moderate symptoms, grade 3 is severe symptoms, grade 4 is life-threating symptoms, and grade 5 is death from radiation-induced symptoms. These grades are referenced to describe outcomes in some of the below studies.

A meta-analysis of 14 retrospective studies by Hasan et al. (84) comparing GTR alone vs. GTR plus adjuvant radiotherapy (RT) in patients with atypical meningioma reported significantly higher 5 year recurrence rates in those receiving GTR alone compared with those also receiving radiation therapy (33 vs. 15%; *p* = 0.005). Of the patients who experienced recurrence, recurrence occurred an average of 8 months later in those treated with radiation (39.5 vs. 31.5 months; *p* = 0.014). In the five studies reporting survival rates, there were similar 5-year overall survival rates in those with GTR vs. GTR plus RT (89.7 vs. 89.4%; *p* = 0.95). Radiation-induced toxicities occurred in <10% of patients with severe toxicities reported in <10%, which included radiation necrosis, visual impairment, and cognitive dysfunction. No life-threatening radiation-induced toxicities were reported. Results support that the benefits of adjuvant radiation may outweigh the risks; however, the authors caution that due to the small number of retrospective studies available for their meta-analysis, no clear recommendations can be made (84).

Another meta-analysis by Graffeo et al. (64) with seven studies plus data from the author's institution comparing GTR alone vs. GTR plus RT in patients with atypical meningioma found a trend toward lower 5 year recurrence rates in patients treated with radiation; however, this did not reach statistical significance (12 vs. 19%; *p* = 0.2). Additionally, in the five studies with survival data, there was a trend toward improved overall survival in patients treated with radiation; however, this also did not reach statistical significance (96 vs. 87%; *p* = 0.4). Radiation-induced toxicities occurred in <10% of patients, and they reported only 1 life-threatening toxicity (85). Similar to Hasan et al., the results support that benefits of adjuvant radiation may outweigh risk, but due to the small number of retrospective studies available for analysis, these authors also reported that no definitive recommendations can be made.

A recent phase II trial (RTOG 0539), investigated outcomes of recurrent grade I and completely resected grade II meningiomas treated with adjuvant RT using a standard dose of 54 Gy. They observed at 93.8% PFS at 3 years, which was significantly higher than historical controls (*p* = 0.003). They also described a 4.1% recurrence rate and 96% overall survival rate at 3 years with low rates of radiation-induced toxicities (97). Another phase II trial (EORTC 22042–26042), evaluated atypical meningiomas following complete resection treated with adjuvant radiotherapy using a high-dose of 60 Gy. They reported a 90% PFS at 3 years and a 96.4% survival rate over the same time period (98). These phase II studies are the first prospective studies to report a benefit to RT for atypical meningiomas following complete resection.

Currently, there is an international, multicenter, randomized control phase III randomized control trial (ROAM-EORTC 1308) comparing adjuvant RT with active monitoring in patients with atypical meningioma follow gross total resection. Patients randomized to the radiosurgery arm will receive 60 Gy in 30 fractions over 6 weeks. This will be the first randomized control trial comparing these two management approaches for patients with atypical meningiomas. Hopefully, the results of this study will clarify the controversy regarding adjuvant radiotherapy in these patients and guide clinical decision making (97).

Additionally, the optimal radiation dose also remains undefined. Historically, radiation doses ranging from 50 to 60 Gy administered in 1.8–2.0 Gy fractions to the tumor bed and any residual tumor with a margin ranging from 0.5 to 2.0 cm (64, 84). Atypical meningiomas are usually treated with a median of 54 Gy and anaplastic meningioma treated with high doses with a median of 60 Gy (99). Although doses from 50 to 70 Gy have been used, there is evidence from several retrospective studies suggesting that higher doses may improve patient outcomes (34, 65, 88, 93, 100, 101). Recurrent meningiomas may even be treated with higher doses at ranges of 65–70 Gy (13).

Due to advancements in radiation technique, several new options have emerged for delivery of radiation to meningioma. In addition to conventional fractionated photon radiotherapy, these modalities include stereotactic radiosurgery (SRS), fractionated stereotactic conformal radiotherapy (FSRS), intensity modulated photon radiation therapy (IMRT), and particle therapies with protons or carbon ions. These methods and the evidence for their use in treating meningiomas are described below (Table 4).

**Table 4:** Summary of radiation treatments types.

	**RT**	**SRS**	**SRT**	**IMRT**	**PBT**	**CIRT**
Radiation type	Photon	Photon	Photon	Photon	Proton beam	Ion beam
Total Dose	50–70 Gy	12–20 Gy	15–35 Gy	54–60 Gy	45–66 Gye	30–48 Gy
Fractions	~30	1	3–6	~30	15–30	10–16
Dose/fraction	1.8–2 Gy	12–20 Gy	Variable; over 3–6 fractions	1.8–2.0 Gy	1.8–3 Gye	~3 Gye
Pros	Well-studied; accessibility	Stereotactic precision; Single treatment	Stereotactic precision; higher doses than SRS	Precise targeting; conformal dose	Lower toxicity; better dose distribution	Lower toxicity; better dose distribution
Cons	Higher toxicity	Higher risk of edema	Needs further study	Needs further study	Higher cost; accessibility	Higher cost, accessibility
Indications	Primary;^**^ residual; recurrent	Residual; recurrent	Residual; recurrent	Primary;^**^ residual; recurrent	Primary;^**^ residual; recurrent	Primary;^**^ residual; recurrent

*CIRT, Carbon ion radiotherapy; PBT, Proton beam therapy; IMRT, Intensity modulated photon therapy; RT, Radiotherapy; SRS, Stereotactic radiosurgery; SRT, Stereotactic radiotherapy*.

** *Primary refers to primary tumor following surgical resection*.

### Stereotactic Radiosurgery (SRS) and Fractionated Stereotactic Radiosurgery (FSRS)

SRS delivers a single high dose of precisely targeted radiation. It has been increasingly utilized over time due to its high rates of local tumor control, improved dose conformity with better dose conformity and sparing surrounding normal tissue from extraneous radiation, and convenience of being delivered in a single fraction (102–105). It is generally used in atypical and anaplastic meningiomas with residual or recurrent disease. Treatment doses typically range from 12 to 20 Gy (67, 81, 90, 91, 106–108). However, one study of SRS for recurrent atypical and anaplastic meningiomas reported worse tumor control with doses <20 vs. 20 Gy with PFS at 5 years of 29 and 63%, respectively (106). In addition to lower radiation dose, other factors associated with increased recurrence and overall worse outcomes following SRS are larger tumor volumes and suboptimal coverage (67, 81, 90, 91, 106–108).

However, several retrospective studies have described SRS to be associated with high rates of symptomatic perilesional edema ranging from 2.5 to 50%. Risk factors associated with developing perilesional edema include prior radiation treatment, larger tumor volume, higher tumor grade, and parasagittal location (103–105). Thus, due to this risk of edema, there has been more interest in treating meningiomas with FSRS instead.

FSRT delivers several fractions of higher radiation doses while maintaining stereotactic precision. Several retrospective studies of FSRS have described delivery of radiation doses of 15–35 Gy over 3–6 fractions in meningiomas with similar local tumor control and slightly lower rates of perilesional edema ranging from 2.7 to 26% compared with SRS (103, 105, 109–112). One study reported that rates of perilesional edema rose as radiation dose per fraction increased with rates of 2.7, 8.8, and 11.9% with fractions of 6 Gy or less, 7–14 Gy, and 15 Gy or higher, respectively (105). However, most authors agree that larger, prospective trials should be conducted to better evaluate this modality.

### Intensity-Modulated Photon Radiotherapy (IMRT)

IMRT is an advanced form of radiotherapy that delivers a conformal isodose of photons to the target. Computer controlled linear accelerators allows radiation dose to more precisely conform to the three-dimensional volume of the tumor by modulating the intensity of the radiation beam delivered to the tumor. Furthermore, this precise delivery allows IMRT to use higher radiation doses targeted to the tumor while minimizing radiation exposure to the surrounding normal brain structures.

A phase II trial of IMRT administered radiation doses ranging from 54 to 60 Gy in 30 fractions for treatment of incompletely resected atypical meningiomas, anaplastic meningiomas regardless of extent of resection, and recurrent meningiomas. The authors reported an overall 3-year PFS of 59% and overall survival of 79%. With the exception of one grade 5 radiation-induced toxicity of necrosis, the other acute and late toxicities were limited to grade 1–3. The authors concluded that overall IMRT was safe and effective in atypical, anaplastic, and recurrent meningiomas, and this therapy deserves further study in these patients (113).

### Particle Radiation Therapies

Unlike conventional photon radiation, particle therapy uses protons or carbon ions to deliver radiation. Compared with photons, protons and carbon ions are more homogeneous and have better dose conformity, allowing more precise delivery of higher radiation doses to tumor cells while limiting radiation to surrounding healthy brain structures. Several studies have reported less radiation-induced toxicity with particle therapy than with photon radiation. Most studies describe predominantly skin irritation and alopecia with minimal to no acute or late severe toxicity (114–118). Re-irradiation with photons is challenging due to the surrounding healthy tissue's limited tolerance to more radiation; however, particle therapy has been described as safe and effective for re-irradiation in recurrent or progressive meningiomas (114). Disadvantages to both proton and carbon ion therapies are limited availability and higher cost than photon radiation therapies. Several studies have been conducted to explore whether the benefits of these therapies outweigh the increased expense of these therapies, but results have been variable (114, 119).

A study comparing proton beam therapy (PBT) alone (56 GyE in 1.8–2 GyE daily fractions), IMRT (50 in 2 Gy daily fractions) with carbon ion radiotherapy (CIRT) boost (18 with 3 Gy daily fractions), IMRT (median 56 in 1.8–2 Gy daily fractions), and fractionated SRT (56 in 1.8–2 Gy daily fractions) found tumor shrinkage and local control at 1 and 2 years follow up was independent of radiation modality. Instead, tumor grade and extent of resection appeared to be the determining factors of tumor shrinkage and local control (115).

Another study comparing PBT with IMRT for atypical, anaplastic, and recurrent meningiomas reported similar dose conformity to the tumor volume but observed significantly less extraneous radiation exposure to surrounding structures with PBT. Thus, higher radiation doses were prescribed for PBT (66 in 2.2 Gy fractions) than for IMRT (54 in 1.8 Gy fractions) with fewer radiation induced tumors. Thus, the authors reported that higher radiation doses allowable with PBT may improve local tumor control and reduce radiation-induced toxicities (120).

In a recent systematic review of ion therapies in atypical and anaplastic meningiomas, PBT and CIRT demonstrated higher rates of PFS compared with conventional photon radiation. Comparing ion therapies, PBT had superior PFS compared with CIRT (121). However, another study reported that CIRT has better dose conformity to tumor volume with reduced extraneous radiation exposure to surrounding brain structures than both PBT and IMRT (117). In a phase I/II trial of CIRT (18 Gy) boost with either FSRT or IMRT (54 Gy) for atypical and anaplastic meningiomas, addition of CIRT appeared to be well tolerated and potentially beneficial to these patients. The authors conclude, however, that a larger prospective trial is needed to corroborate these findings (116).

## Chemotherapy and Other Medical Therapies

Chemotherapy and other systemic therapies have demonstrated limited clinical efficacy in treating meningiomas (122). Although marginal, interferon-alpha, somatostatin receptor antagonists, and VEGF receptor inhibitors are the only FDA-approved agents providing any benefit to these patients. Currently, these options are used for salvage therapy for meningiomas recurrence or progression following surgery and radiation that have become refractory or no longer amenable to these standard treatment options.

Several chemotherapeutic agents have been studied for meningioma with minimal clinical efficacy. Hydroxyurea has been studied in many other cancers, and it is one of the most studied chemotherapeutic agents in meningioma. In preclinical trials, hydroxyurea reduced meningioma growth (123, 124), however, it has failed to provide similar results in clinical trials and other human studies (125–127). Other chemotherapeutic agents, including temozolomide, irinotecan, and combination therapy with cyclophosphamide, Adriamycin, and vincristine, have not shown benefit in treating meningiomas (128–130).

Interferon-alpha is an immunomodulating agent demonstrating slight therapeutic benefit in recurrent meningiomas not amenable to resection. Several studies demonstrated stabilization of tumor growth, and a phase II study of recurrent meningiomas reported a slight improvement in PFS at 12 weeks without improvement in overall survival rates (131–133).

As mentioned above, overexpression of somatostatin receptors is associated with more aggressive tumors and higher recurrence rates. Thus, several somatostatin receptor inhibitors have been studied in recurrent meningiomas with questionable therapeutic effects. In one study using a long-acting inhibitor sandostatin, the authors observed a slight improvement in PFS and overall survival at 6 months (134), but other phase II clinical trials using sandostatin, octreotide, or other somatostatin receptor inhibitors have demonstrated minimal efficacy and not reported similar results (135, 136). Other hormone receptor inhibitors, including antiestrogen and antiprogesterone agents, have not demonstrated clinical benefits (137–142).

Similar to other neoplasms, meningiomas often overexpress VEGF, PDGF, EGF, and other growth factor receptors. Overexpression is hypothesized to promote tumor growth. Thus, a variety of therapies using monoclonal antibodies or small molecule kinase inhibitors targeting one or more of these receptors have been studied in recurrent meningiomas. Unfortunately, studies using these targeted therapies alone or in combination have demonstrated limited or no success in treatment of meningioma (143–149).

Several studies using bevacizumab, a monoclonal antibody against the VEGF receptor, have reported mild improvement in PFS in patients with recurrent meningiomas (150–152). Despite slight benefit, the overall clinical efficacy remains poor with one systematic review of bevacizumab in recurrent meningioma reporting median PFS of 15.3 months in recurrent atypical and 3.7 months in anaplastic meningiomas (150). A phase II trial of bevacizumab plus everolimus (an mTOR inhibitor) reported PFS similar to prior studies of bevacizumab alone (148).

Sunitinib is a small molecule kinase inhibitor that targets both VEGF receptor and PDGF receptor. In phase II clinical trials of sunitinib for recurrent and progressive atypical and anaplastic meningiomas, there was a PFS of 42% at 6 months, which was an improvement from reported natural history PFS of 5–30% at 6 months. Toxicity, however, was a concern with 60% of patients experiencing a severe or life-threatening event. The authors recommend that sunitinib warrants further investigation with a larger, randomized trial to better characterize the efficacy of sunitinib in this population of patients (143).

Erlotinib and gefitinib are both small molecule kinase inhibitors of EGF receptor that have been studied in phase II trials for recurrent meningioma. Although these therapies were well tolerated, they did not improve PFS or overall survival of these patients (144). Similarly, in a phase II trial of imatinib, a small molecule kinase inhibitor of PDGF receptor, in recurrent meningioma, the therapy was well tolerated, but did not prolong PFS in these patients (149).

## Discussion

**Figure 3:**
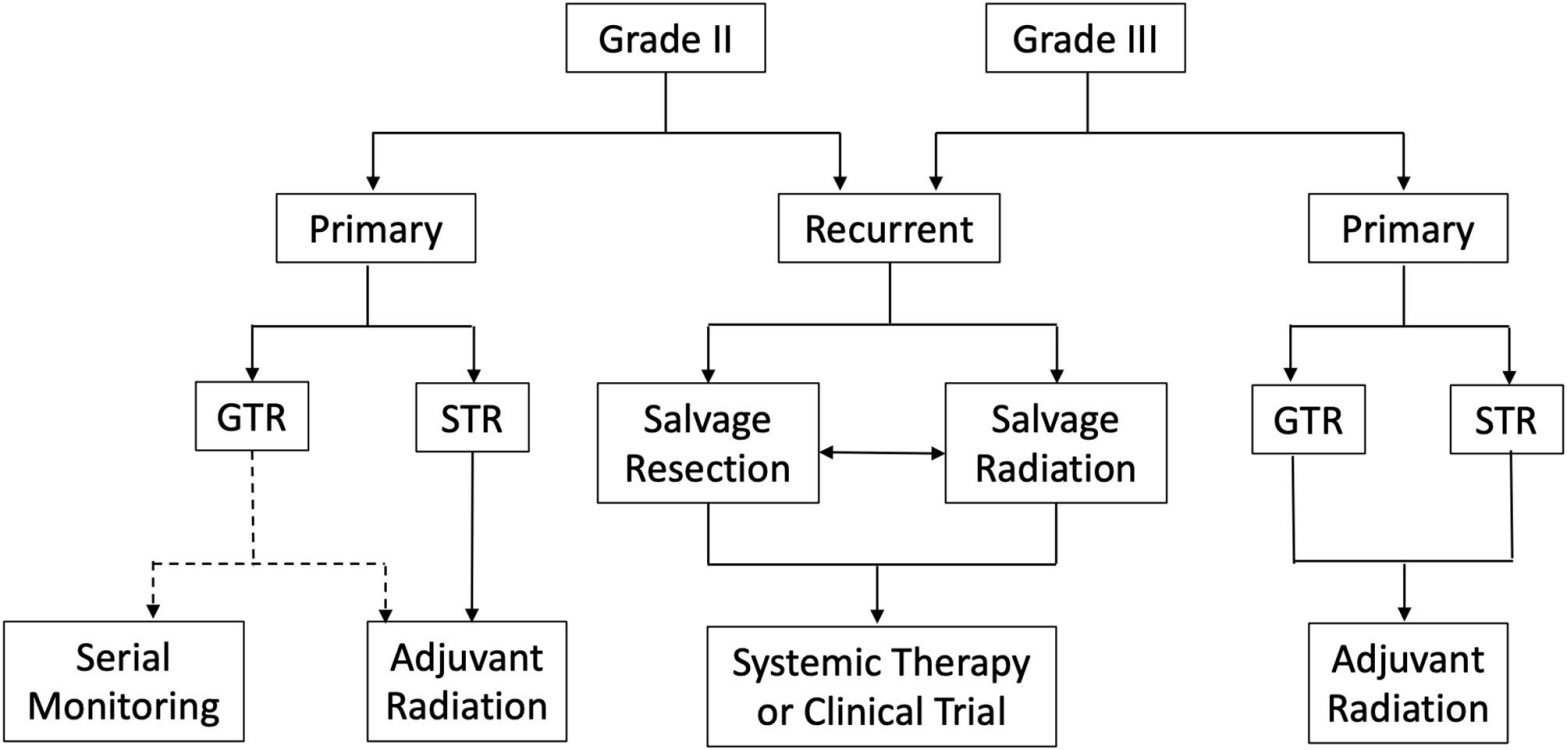
Summary of management strategies for atypical and anaplastic meningiomas. ^*^GTR, Gross total resection; STR, Subtotal resection; Dotted line represents lack of consensus regarding serial monitoring vs. adjuvant radiation following complete resection of WHO grade II meningioma.

Atypical and anaplastic meningiomas remain challenging to treat. Currently, the standard of care is maximum safe resection followed by adjuvant radiation for grade III and incompletely resected grade II meningiomas. However, controversy surrounds the role of adjuvant radiation for completely resected grade II meningiomas (Figure 3). Advocates argue adjuvant radiation reduces recurrence and lengthens progression free survival in those who recur; conversely, opponents contend adjuvant radiation does not reduce recurrence and introduces further costs and potential harm from possibly unnecessary radiation. Moreover, the literature offers inconsistent and ultimately inconclusive data. However, as mentioned above, ROAM-EORTC 1308 is a phase III randomized clinical trial investigating adjuvant RT vs. active monitoring in patients with atypical meningioma following gross total resection. Hopefully, the results of this study will clarify this controversy and provide insight into clinical decision making (97).

Despite maximum treatment, atypical and anaplastic meningiomas have a strong proclivity for recurrence. Accumulating mutations over time, recurrent tumors behave more aggressively and often become refractory or no longer amenable to further surgical resection or radiation. Chemotherapy and other medical therapies are available as salvage treatment once standard options are exhausted; however, efficacy of these agents remains limited. Furthermore, accurate risk stratification remains an obstacle. Across all grades, meningiomas exhibit a spectrum of aggressive behavior only partially predicted by histological criteria alone. Clinically, this translates into difficulty predicting prognosis and determining the optimal management approach.

Despite these challenges, however, advances in oncologic research and technology provide hope by uncovering new and informative genetic mutations, aberrant signaling pathways, and protein biomarkers associated with tumor behavior and recurrence risk. Understanding the pathophysiology and molecular biology of meningiomas is critical in more adequately predicting prognosis, discovering novel therapeutic approaches, and tailoring treatment to individual patients and the biology of their meningiomas.

## Author Contributions

All authors listed have made a substantial, direct and intellectual contribution to the work, and approved it for publication.

## Conflict of Interest

The authors declare that the research was conducted in the absence of any commercial or financial relationships that could be construed as a potential conflict of interest.
